# Multifrequency controlled synchronization of four inductor motors by the fixed frequency ratio method in a vibration system

**DOI:** 10.1038/s41598-023-29603-y

**Published:** 2023-02-11

**Authors:** Lei Jia, Chun Wang, Ziliang Liu

**Affiliations:** School of Mechanical Engineering, Shenyang Ligong University, Shenyang, 110159 China

**Keywords:** Mechanical engineering, Nonlinear phenomena

## Abstract

In this article, multifrequency controlled synchronization of four inductor motors by the fixed frequency ratio method in a vibration system is investigated. The electromechanical coupling dynamical model of the vibrating system is established. The synchronous condition of the vibrating system is obtained with the small parameter method. Through the theoretical derivation and numerical simulation, multifrequency self-synchronization of four induction motors in the vibration system can’t be realized. To achieve the purpose of multifrequency synchronization motion, the method of multifrequency controlled synchronization is proposed, and a fuzzy PID controlling method is introduced. The stability of the controlling system is certified by the Lyapunov criterion. An arbitrariness of the proposed controlling method which is applied to the vibration system is presented. To certify the accuracy of the theory and simulation, a vibrating test bench is constructed. Some experiments are operated to validate the effectiveness and the proposed controlled synchronization method.

## Introduction

With the development of the economy, the pursuit of interest appears to be particularly important in the industry production. For achieving this target, many technologies corresponding to it are presented. In the meanwhile, vibrating machines as a branch in the industry are investigated for benefits on the agriculture, for example, the vibrating screen, the vibrator feeder and so on^1–4^. These kinds of vibrating machines are usually structured by two patterns in the industry. One type as a forced synchronization is realized by belts, gears etc. They can implement the same or different speeds among inductor motors. The other type is based on the self-synchronization theory which is firstly proposed by Blekhman^5,6^. In their research, the dynamic model is combined with the multiscale method which is an asymptotic analytic method based on the average method. By utilizing different time scales, they divide the vibrating motion into two kinds of processes which are respective fast and slow processes. The fast one is relative to the motor speed and the slow one is relative to the phase. Thus, two eccentric rotors (ERs) driven by induction motors realize the self-synchronization on the opposite directions. Obviously, the vibrating machines can be realized with more simple structure and fewer cost by the self-synchronization theory. Based on the former results, many researchers are attracted in this field and it acquires a rapid development. Wen et al.^7^ analyses the feature of vibrating system based on a high coupling dynamical model. In addition, they derived synchronous and stability conditions of the vibrating system with the Hamilton criterion. Zhao et al.^8,9^ establish the electromechanical coupling dynamic model and converts the problem of synchronous condition into an existence of the eigenvalue with the small parameter average method. They not only realize the self-synchronization motion of two motors in the opposite directions but also in the same directions. The researches above are established in one single rigid body. Zhang et al.^1,10–13^ present the theory of self-synchronization with multi motors (more than two motors). In their research, the dynamical model is established based on a rigid body. With the synchronization condition and synchronization criteria of self-synchronization, the characteristic analysis of dynamical model is given. Their research results present that the self-synchronization of vibrating system with three motors can’t obtain a superposed amplitude and this phenomenon don’t realize the zero differences among three motors. The synchronization problems above are all based on the same frequency of motors. The synchronization problem with different frequency is presented by Inoue Junki chi^14^. In their work, four motors are symmetrically installed on a vibrostand along the vertical axis rather than horizontal axis. And they use this asymmetrical feature to realize the multi-frequency synchronization. However, the mothed in Ref.^14^ can only realize one synchronous state with the fixed dynamical model. This result can’t satisfy the needs of industry.

The self-synchronization motion which can realize the zero difference between two motors should satisfy the synchronization condition and synchronization criteria. And this result depends on the dynamical feature of the vibrating system. To solve this problem, controlling methods are introduced in the vibrating system. Kong et al.^15,16^ introduce the controlling strategy and method into self-synchronization motion and realize the controlled synchronization motion. In their research, a master–slave controlling strategy and an adaptive sliding mode controlling method is presented to be used in the dynamical model of the vibrating system. With this method, the differences of motors which are not zero in the self-synchronization can be realized to zero finally. Besides the method above, Ishizaki et al.^17^ use the cross-coupling controlling method to realize the synchronization with dual servo systems. Aiming at the dual linear motor system, Lin et al.^18^ apply intelligent complementary sliding mode controlling method to implement cross-coupled synchronous. Attention to different frequency of motors in the synchronization motion, Jia et al.^19,20^ propose the adaptive fuzzy PID method and realize the multifrequency controlled synchronization motion. Tian et al.^21^ illustrate the fast and robust estimation for positions and velocities by using a generalized modulating functions method. Balthazar et al.^22^ gives the research of self-synchronization of four non-ideal exciters. Nanha Djanan et al.^23,24^ study the self-synchronization motion on a rectangular plate.

From illustrations above, the purpose of realizing the synchronization motions is to increase the amplitudes of the vibrating system based on the dynamical feature. And this result can be converted to realize the zero differences between motors. However, the multi-frequency synchronization can only be realized with integer frequency (2 or 3 times). For solving this problem, multifrequency controlled synchronization of four inductor motors by fixed frequency ratio method is proposed and the main structure in this article is provided. In section "Mathematical model and synchronization analysis", the dynamical model of the vibrating system with four motors is established. And then synchronization condition and criteria of the vibrating system are both obtained in the least common multiple cycle with the small parameter method. In section "Design of the controlling system", the adaptive fuzzy PID controlling method is introduced in the vibrating system based on a master–slave controlling strategy and the stability of the controlling system is certified by Lyapunov theory. For a legible illustration, some numerical simulations and experiments are shown in section "Numerical and experimental results with discussions" and the conformity between simulation and experiment are listed. In section "Conclusions", some conclusions about this article are given.

## Mathematical model and synchronization analysis

### Mathematical model of the vibrating system

**Figure 1:**
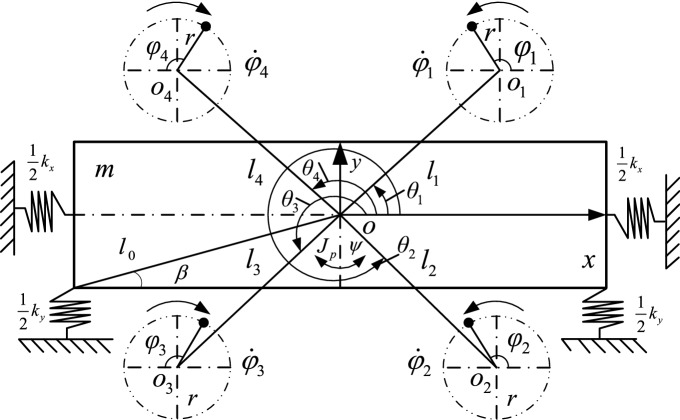
Mathematical model of the vibrating system.

In this section, the mathematical model of the vibrating system is shown in Fig. 1, which is established from bottom to the top. All the symbols are listed in Table 1. One rigid frame is connected on the foundation with four springs which are symmetrically distributed along coordinate axis. Four squirrel-cage inductor motors divided into two groups are fixed on the frame symmetrically. Motor 1 and 4 as one group have the same frequency and the other group are consisted of motor 2 and 3 of which frequency are different form the front group. Each motor is installed by four bolts through circular holes on the frame.

**Table 1:** The nomenclature table of the symbols.

symbol	explanation
*m*_*i*_	The mass of each ERs
*J*_*p*_	The moment of inertia of the rigid body
*J*	The moment of inertia of the vibration system
*k*_*x*_, *k*_*y*_, *k*_*ψ*_	The damping coefficients of the vibration system in the *x*, *y* and *ψ* directions
*r*	The eccentric radius of the four motors
*M*	The mass of the total vibration system
*f*_*x*_, *f*_*y*_, *f*_*ψ*_	The stiffness coefficients of the vibration system in the *x*, *y* and *ψ* directions
*l*_1_,* l*_2_,* l*_3_,* l*_4_	The distance between the center of the body and the rotating center of motors
*J*_*i*_	The moment of inertia of the inductor motor
*d-*,*q-*	The d- and q- axes in rotor field-oriented coordinate
*L*_*s*_	Self-inductance of the stator
*L*_*r*_	Self-inductance of the rotor
Subscript *s*	Stator
Subscript* r*	Rotor
*L*_*m*_	Mutual inductance of the stator and rotor
$\phi_{sd}$	The flux linkages of the stator in the *d*- axis
$\phi_{sq}$	The flux linkages of the stator in the *q*- axis
$\phi_{rd}$	The flux linkages of the rotor in the *d*- axis
$\phi_{rq}$	The flux linkages of the rotor in the *q*- axis
*R*_*s*_	The stator resistance
*R*_*r*_	The rotor resistance
*i*_*sd*_	The current of stator in the *d*- axis
*i*_*sq*_	The current of stator in the *q*- axis
*i*_*rd*_	The current of rotor in the *d*- axis
*i*_*rq*_	The current of rotor in the *q*- axis
*ω*	The mechanical speed
*ω*_*s*_	The synchronous electric angular speed
$\dot{\phi }_{sd}$, $\dot{\phi }_{sq}$, $\dot{\phi }_{rd}$,$\dot{\phi }_{rq}$	Derivation of $\phi_{sd}$,$\phi_{sq}$,$\phi_{rd}$,$\phi_{rq}$
*σ*	The leakage factor
*Tr*	A rotor time constant
*u*_*sd*_	The voltage of stator in the d- axis
*u*_*sq*_	The voltage of stator in the q- axis
*u*_*rd*_	The voltage of rotor in the d- axis
*u*_*rq*_	The voltage of rotor in the q- axis
*n*_*p*_	The number of pole-pairs of the induction motor
$\bullet^{ * }$	The given values or obtained from the given values
$\omega_{1}$,$\omega_{2}$,$\omega_{3}$,$\omega_{4}$	The speeds of four motors
$\varphi_{1}$,$\varphi_{2}$,$\varphi_{3}$,$\varphi_{4}$	The phases of four motors
${\uptheta }$	Synchronization electric angle

As shown in Fig. 1, *o* is center point of the frame and *o*_*i*_ (*i* = 1, 2, 3, 4) are respectively shaft points of four inductor motors. $oo_{i} = l_{i}$ (*i* = 1, 2, 3, 4) are respectively the distances between center point of the frame *o* and shaft points of four inductor motors *o*_*i*_. *m* is the quality of the frame and four inductor motors. *m*_0_ is the full quality of each ERs and *m*_*i*_ (*i* = 1, 2, 3, 4) are respectively the actual quality of four ERs. *r* is the radius of four motors. $\varphi_{i} (i = 1,2,3,4)$ are initial phase angle of inductor motors. $\theta_{i} (i = 1,2,3,4)$ are position angle of four ERs.$J_{p}$ is rotational inertia of the frame. $\psi$ is the swing angle of the vibrating system. Thus, the differential equation of the vibrating system based on the Lagrange equation can be expressed as

1 $$ \frac{d}{dt}\left( {\frac{\partial L}{{\partial {\dot{\mathbf{q}}}}}} \right) - \frac{\partial L}{{\partial {\mathbf{q}}}} = {\mathbf{Q}} $$

where $L = T - V$. *L* is the Lagrange function. *T* and *V* are respectively the kinetic energy and potential energy. ***Q*** and ***q*** respectively represent generalized force and generalized coordinates. ${\mathbf{Q}} = ( - f_{x} \dot{x}, - f_{y} \dot{y}, - f_{\psi } \dot{\psi },T_{e1} - f_{1} \dot{\varphi }_{1} ,T_{e2} - f_{2} \dot{\varphi }_{2} ,T_{e3} - f_{3} \dot{\varphi }_{3} ,T_{e4} - f_{4} \dot{\varphi }_{4} )^{{\text{T}}}$, ${\mathbf{q}} = (x,y,\psi ,\varphi_{1} ,\varphi_{2} ,\varphi_{3} ,\varphi_{4} )^{{\text{T}}}$.

2 $$ T = m(\dot{x} + \dot{y})^{2} /2 + J_{p} \dot{\psi }^{2} /2 + \sum\limits_{i = 1}^{4} {m_{0} } {\dot{\mathbf{x}}}_{i}^{{\text{T}}} {\dot{\mathbf{x}}}_{i} /2 + \sum\limits_{i = 1}^{4} {J_{i} } \dot{\varphi }_{i}^{2} /2 $$

In Eq. (2), $${\mathbf{x}}_{i} = \left( \begin{gathered} x \hfill \\ y \hfill \\ \end{gathered} \right) + \left( {\begin{array}{*{20}c} {\cos \psi } & { - \sin \psi } \\ {\sin \psi } & {\cos \psi } \\ \end{array} } \right)\left( \begin{gathered} l_{i} \cos \theta_{i} + \tau_{i} r\cos \varphi_{i} \hfill \\ l_{i} \sin \theta_{i} + r\sin \varphi_{i} \hfill \\ \end{gathered} \right)$$.

3 $$ V = k_{x} x^{2} /2 + k_{y} y^{2} /2 + k_{\psi } \psi^{2} /2 $$

Combined Eq. (1) with Eqs. (2) and (3), the electromechanical coupling dynamical model of the vibrating system can be obtained.

4 $$ \begin{gathered} \hfill \\ \begin{array}{*{20}c} {M\ddot{x} + f_{x} \dot{x} + k_{x} x = \sum\limits_{i = 1}^{4} {\tau_{i} } m_{i} r(\dot{\varphi }_{i}^{2} \cos \varphi_{i} + \ddot{\varphi }_{i} \sin \varphi_{i} )} \\ {M\ddot{y} + f_{y} \dot{y} + k_{y} y = \sum\limits_{i = 1}^{4} {m_{i} r(\dot{\varphi }_{i}^{2} \sin \varphi_{i} - \ddot{\varphi }_{i} \cos \varphi_{i} )} } \\ {J\ddot{\psi } + f_{\psi } \dot{\psi } + k_{\psi } \psi = \sum\limits_{i = 1}^{4} {m_{i} rl_{i} } [\dot{\varphi }_{i}^{2} \sin (\varphi_{i} - \tau_{i} \theta_{i} ) - \ddot{\varphi }_{i} \cos (\varphi_{i} - \tau_{i} \theta_{i} )]} \\ {J_{i} \ddot{\varphi }_{i} + f_{i} \dot{\varphi }_{i} = T_{ei} - T_{Li} } \\ \end{array} ,\quad \tau_{i} = \left\{ \begin{gathered} + 1,{\kern 1pt} {\kern 1pt} {\kern 1pt} {\kern 1pt} {\kern 1pt} {\kern 1pt} i = 1,2 \hfill \\ - 1,{\kern 1pt} {\kern 1pt} {\kern 1pt} {\kern 1pt} {\kern 1pt} i = 3,4 \hfill \\ \end{gathered} \right. \hfill \\ \end{gathered} $$

where $M = m + \sum\nolimits_{i = 1}^{4} {m_{i} }$ is the total mass of the vibrating system, $J = Ml_{e}^{2} \approx J_{p} + \sum\nolimits_{i = 1}^{4} {m_{i} } (l_{i}^{2} + r^{2} )$ is the equivalent rotational inertia of the vibrating system. $l_{e}$ is the equivalent rotational radius. $J_{i} = m_{i} r^{2} (i = 1,2,3,4)$ are respectively rotational inertia of four motors. $f_{x}$, $f_{y}$ and $f_{\psi }$ are respectively the damping coefficients of vibrating system in $x$, $y$ and $\psi$ directions, $f_{\psi } = f_{x} l_{y}^{2} + f_{y} l_{x}^{2}$. $k_{x}$, $k_{y}$ and $k_{\psi }$ are respectively the stiffness coefficients of vibrating system in $x$, $y$ and $\psi$ directions, $f_{\psi } = f_{x} l_{y}^{2} + f_{y} l_{x}^{2}$. $f_{i} (i = 1,2,3,4)$, $T_{ei} (i = 1,2,3,4)$, and $T_{Li} (i = 1,2,3,4)$ are respectively damping coefficients, electromagnetic torques and load torques of four motors. The item *T*_*L*_ in Eq. (4) can be derived as Eq. (5).

5 $$ T_{Li} = m_{i} r[\ddot{y}\cos \varphi_{i} - \tau_{i} \ddot{x}\sin \varphi_{i} + l_{i} \ddot{\psi }\cos (\varphi_{i} - \tau_{i} \theta_{i} ) + \tau_{i} l_{i} \dot{\psi }^{2} \sin (\varphi_{i} - \tau_{i} \theta_{i} )] $$

Aiming to illustrate the item *T*_*e*_ in the electromechanical coupling dynamical model, the model of inductor motor is given. In this article, the ERs are driven by squirrel-cage inductor motors. With the feature of this kind of inductor motor, its inner rotor windings short-circuit. Thus, $u_{rd} = u_{rq}$. When the motor is at a stable state, $\phi_{rd}$ = constant and $\phi_{rq}$ = 0. According to document^25^, the model of the inductor motor can be expressed as Eq. (6).

6 $$ \begin{gathered} L_{ks} di_{sd} /dt = u_{sd} - R_{ks} i_{sd} + R_{r} L_{m} /L_{r}^{2} \phi_{rd} + \omega_{s} L_{ks} i_{sq} \hfill \\ L_{ks} di_{sd} /dt = u_{sq} - R_{ks} i_{sq} - L_{m} /L_{r} \phi_{rd} \omega - \omega_{s} L_{ks} i_{sd} \hfill \\ d\phi_{rd} /dt = 1/T_{r} (L_{m} i_{sd} - \phi_{rd} ) \hfill \\ d{\uptheta }/dt = L_{m} i_{sq} /T_{r} \phi_{rd} + \omega \hfill \\ T_{e} = 3L_{m} \phi_{rd} i_{sq} /2L_{r} \hfill \\ \end{gathered} $$

where, *s* and *r* respectively represent stator and rotor. *d-* and *q-* represent *d*- axis and *q*- axis in the rotating coordinate frame. *i*, *u* and *R* respectively represent current, voltage and resistance. *L*_*s*_ and *L*_*r*_ are respectively represent self-inductance of the stator and rotor. *L*_*m*_ is mutual inductance of the stator and rotor. *T*_*r*_ is rotor time constant, $T_{r} = L_{r} /R_{r}$. *L*_*ks*_ is leakage inductance of the stator, $L_{ks} = L_{s} - L_{m}^{2} /L_{r}$. *R*_*ks*_ is equivalent resistance of the stator, $R_{ks} = R_{s} + L_{m}^{2} R_{r} /L_{r}^{2}$. ${\uptheta }$ represents the synchronous flux-linkage angle, ${\uptheta } = \int {(\omega + \omega_{s} )} dt$. $\omega$ represents the mechanical angular velocity. $\omega_{s}$ represents the synchronous electrical angular velocity, $\omega_{s} = L_{m} i_{sq} /\phi_{rd} T_{r}$.

**Figure 2:**
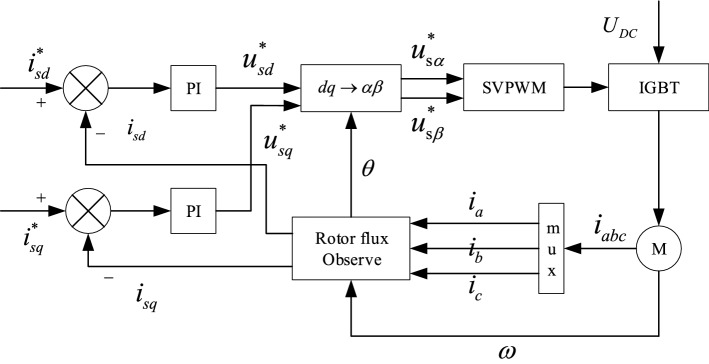
RFOC: rotor flux-oriented control.

To realize the controlled synchronization, the vector control method is introduced in the controlling system. And the rotor flux-oriented control (RFOC) is shown in Fig. 2 to illustrate the controlling system. In Fig. 2, signs with “$*$” are initial values and in Eq. (5), $L_{m}$ and $\phi_{rd}$ are given values. $i_{sd}$ can be calculated by formulation $i_{sd} = \phi_{rd} /L_{m}$. Therefore, the item *T*_*e*_ is related with the feedback values $i_{sq}$.

### Synchronization analysis of the multifrequency self-synchronization

To show the stability analysis of the vibrating system clearly, the illustration of the dynamic model should be given firstly. In this model, motor 1 and motor 4 rotated in the opposite direction as shown in Fig. 1 firstly. When the two motors realize the stable self-synchronization motion with zero phase difference, the stably synchronous state of motor 1 and 4 can be expressed as $\omega_{1} - \omega_{4} = 0$ and $\varphi_{1} - \varphi_{4} = 0$. Then Motor 2 and motor 3 finish the same self-synchronization motion as the condition above. In the same way, the stably synchronous state of motor 2 and 3 can be expressed as $\omega_{2} - \omega_{3} = 0$ and $\varphi_{2} - \varphi_{3} = 0$. Finally, Motor 1 and 4 realize the multifrequency synchronization motion with the fixed frequency ratio method and the stably synchronous state can be presented as $p\dot{\varphi }_{1} - q\dot{\varphi }_{2}$ = 0 and $n\varphi_{1} - \varphi_{2}$ = constant. Where, *p* and *q* are prime numbers, *p*/*q* = *n*. Thus, the speeds of four motors can be presented as

7 $$ \begin{gathered} \omega_{1} { = }\int_{0}^{{T_{1} }} {\dot{\varphi }_{1} } (t)dt/T_{1} \hfill \\ \omega_{2} { = }\int_{0}^{{T_{2} }} {\dot{\varphi }_{2} } (t)dt/T_{2} \hfill \\ \omega_{3} { = }\int_{0}^{{T_{3} }} {\dot{\varphi }_{3} } (t)dt/T_{3} \hfill \\ \omega_{4} { = }\int_{0}^{{T_{4} }} {\dot{\varphi }_{4} } (t)dt/T_{4} \hfill \\ \end{gathered} $$

Taking Eq. (7) into Eq. (4), responses in three directions of the vibrating system can be derived as the Eq. (8).

8 $$ \begin{gathered} x = - r_{m} r[\eta_{1} \cos (\varphi_{1} + \gamma_{x1} )/\mu_{x1} + \eta_{2} \cos (\varphi_{2} + \gamma_{x2} )/\mu_{x2} - \eta_{3} \cos (\varphi_{3} + \gamma_{x3} )/\mu_{x3} \hfill \\ \;\;\;\;\; - \eta_{4} \cos (\varphi_{4} + \gamma_{x4} )/\mu_{x4} ] \hfill \\ y = - r_{m} r[\eta_{1} \sin (\varphi_{1} + \gamma_{y1} )/\mu_{y1} + \eta_{2} \sin (\varphi_{2} + \gamma_{y2} )/\mu_{y2} + \eta_{3} \sin (\varphi_{3} + \gamma_{y3} )/\mu_{y3} \hfill \\ \;\;\;\;\; + \eta_{4} \sin (\varphi_{4} + \gamma_{y4} )/\mu_{y4} ] \hfill \\ \psi = - (r_{m} r/l_{e} )[\eta_{1} r_{l1} \sin (\varphi_{1} - \theta_{1} + \gamma_{\psi 1} )/\mu_{\psi 1} + \eta_{2} r_{l2} \sin (\varphi_{2} - \theta_{2} + \gamma_{\psi 2} )/\mu_{\psi 2} \hfill \\ \;\;\;\;\;\; + \eta_{3} r_{l3} \sin (\varphi_{3} + \theta_{3} + \gamma_{\psi 3} )/\mu_{\psi 3} + \eta_{4} r_{l4} \sin (\varphi_{4} + \theta_{4} + \gamma_{\psi 4} )/\mu_{\psi 4} ] \hfill \\ \end{gathered} $$

where $$\omega_{x}^{2} = k_{x} /M$$, $$\omega_{y}^{2} = k_{y} /M$$, $$\omega_{\psi }^{2} = k_{\psi } /J$$, $$\zeta_{x} = f_{x} /(2\sqrt {k_{x} M} )$$, $$\zeta_{y} = f_{y} /(2\sqrt {k_{y} M} )$$, $$\zeta_{\psi } = f_{\psi } /(2\sqrt {k_{\psi } J} )$$, $$\mu_{xi} = 1 - \omega_{x}^{2} /\omega_{i}^{2}$$, $$\mu_{yi} = 1 - \omega_{y}^{2} /\omega_{i}^{2}$$, $$\mu_{\psi i} = 1 - \omega_{\psi }^{2} /\omega_{i}^{2}$$, $$r_{li} = l_{i} /l_{e}$$, $$\tan \gamma_{xi} = 2\zeta_{x} \omega_{x} /(\mu_{xi} \omega_{i} )$$, $$\tan \gamma_{yi} = 2\zeta_{y} \omega_{y} /(\mu_{yi} \omega_{i} )$$, $$\tan \gamma_{\psi i} = 2\zeta_{\psi } \omega_{\psi } /(\mu_{\psi i} \omega_{i} )$$, $$r_{m} = m_{0} /M$$, $$\eta_{i} = m_{i} /m_{0} (i = 1,2,3,4)$$.

With the small parameter method, the small parameter $\varepsilon$ is introduced in Eq. (4). And then, Eq. (4) can be converted to Eq. (9).

9 $$ \begin{gathered} J_{1} \dot{\overline{\varepsilon }}_{1} \omega_{0} + f_{1} \omega_{0} (p + \overline{\varepsilon }_{1} ) = \overline{T}_{e1} - \overline{T}_{L1} \hfill \\ J_{2} \dot{\overline{\varepsilon }}_{2} \omega_{0} + f_{2} \omega_{0} (q + \overline{\varepsilon }_{2} ) = \overline{T}_{e2} - \overline{T}_{L2} \hfill \\ J_{3} \dot{\overline{\varepsilon }}_{3} \omega_{0} + f_{3} \omega_{0} (q + \overline{\varepsilon }_{3} ) = \overline{T}_{e3} - \overline{T}_{L3} \hfill \\ J_{4} \dot{\overline{\varepsilon }}_{4} \omega_{0} + f_{4} \omega_{0} (p + \overline{\varepsilon }_{4} ) = \overline{T}_{e4} - \overline{T}_{L4} \hfill \\ \end{gathered} $$

where $\omega_{0}$ is the average speed of motors in the self-synchronization. The calculation method of $\overline{T}_{ei} = T_{e0i} - k_{e0i} \overline{\varepsilon }_{i} (i = 1,2,3,4)$ can be obtained from Ref.^15^. Assuming $\dot{\varphi }_{1} = (p + \varepsilon_{1} )\omega_{0}$, $\dot{\varphi }_{2} = (q + \varepsilon_{2} )\omega_{0}$, $\dot{\varphi }_{3} = (q + \varepsilon_{3} )\omega_{0}$, $\dot{\varphi }_{4} = (p + \varepsilon_{4} )\omega_{0}$, $\ddot{\varphi }_{1} { = }\dot{\varepsilon }_{1} \omega_{0}$, $\ddot{\varphi }_{2} { = }\dot{\varepsilon }_{2} \omega_{0}$, $\ddot{\varphi }_{3} { = }\dot{\varepsilon }_{3} \omega_{0}$, $\ddot{\varphi }_{4} { = }\dot{\varepsilon }_{4} \omega_{0}$, the average load torques can be expressed as

10 $$ \begin{gathered} \overline{T}_{L1} = m_{0} r^{2} \omega_{0} (a_{11} \dot{\overline{\varepsilon }}_{1} + a_{14} \dot{\overline{\varepsilon }}_{4} + b_{11} \overline{\varepsilon }_{1} + b_{14} \overline{\varepsilon }_{4} + \kappa_{1} ) \hfill \\ \overline{T}_{L2} = m_{0} r^{2} \omega_{0} (a_{22} \dot{\overline{\varepsilon }}_{2} + a_{23} \dot{\overline{\varepsilon }}_{3} + b_{22} \overline{\varepsilon }_{2} + b_{23} \overline{\varepsilon }_{3} + \kappa_{2} ) \hfill \\ \overline{T}_{L3} = m_{0} r^{2} \omega_{0} (a_{32} \dot{\overline{\varepsilon }}_{2} + a_{33} \dot{\overline{\varepsilon }}_{3} + b_{32} \overline{\varepsilon }_{2} + b_{33} \overline{\varepsilon }_{3} + \kappa_{3} ) \hfill \\ \overline{T}_{L4} = m_{0} r^{2} \omega_{0} (a_{41} \dot{\overline{\varepsilon }}_{1} + a_{44} \dot{\overline{\varepsilon }}_{4} + b_{41} \overline{\varepsilon }_{1} + b_{44} \overline{\varepsilon }_{4} + \kappa_{4} ) \hfill \\ \end{gathered} $$

The coefficient and constant items are all listed in the Online Appendix A.

From the Online Appendix A, it can be known that when two inductor motors are with the same frequency (self-synchronization), the coupling effect exists. Otherwise, there is no coupling effect between two motors. Because motor 1 and motor 4 realize self-synchronization motion, the average phase difference can be expressed as $\varphi_{1} - \varphi_{4} = 2\alpha_{1}$. Based on the same theory, the average phase difference between motor 2 and motor 3 can be expressed as $\varphi_{2} - \varphi_{3} = 2\alpha_{2}$. Take items *T*_*e*_ and *T*_*L*_ into Eq. (10) and expand it at $\alpha_{1}$ and $\alpha_{2}$ respectively. In the meanwhile, omit the higher order non-liner terms and assume $\varepsilon_{5}$ and $\varepsilon_{6}$ to be respectively the small parameter perturbation of two groups of inductor motors. Equation (11) can be obtained.

11 $$ {\mathbf{A}}\user2{\dot{\overline{\varepsilon }}} = {\mathbf{B}}\overline{\user2{\varepsilon }} + {\upupsilon } $$

where, $${\mathbf{A}} = \left( {\begin{array}{*{20}c} {a^{\prime}_{11} } & 0 & 0 & {a^{\prime}_{14} } & 0 & 0 \\ 0 & {a^{\prime}_{22} } & {a^{\prime}_{23} } & 0 & 0 & 0 \\ 0 & {a^{\prime}_{32} } & {a^{\prime}_{33} } & 0 & 0 & 0 \\ {a^{\prime}_{41} } & 0 & 0 & {a^{\prime}_{44} } & 0 & 0 \\ 0 & 0 & 0 & 0 & 1 & 0 \\ 0 & 0 & 0 & 0 & 0 & 1 \\ \end{array} } \right)$$, $${\mathbf{B}} = \left( {\begin{array}{*{20}c} {b^{\prime}_{11} } & 0 & 0 & {b^{\prime}_{14} } & {b^{\prime}_{15} } & 0 \\ 0 & {b^{\prime}_{22} } & {b^{\prime}_{23} } & 0 & 0 & {b^{\prime}_{26} } \\ 0 & {b^{\prime}_{32} } & {b^{\prime}_{33} } & 0 & 0 & {b^{\prime}_{36} } \\ {b^{\prime}_{41} } & 0 & 0 & {b^{\prime}_{44} } & {b^{\prime}_{45} } & 0 \\ {\omega_{0} /2} & 0 & 0 & { - \omega_{0} /2} & 0 & 0 \\ 0 & {\omega_{0} /2} & { - \omega_{0} /2} & 0 & 0 & 0 \\ \end{array} } \right)$$, $${\dot{\overline{\mathbf{\varepsilon }}}} = \left( {\begin{array}{*{20}c} {\dot{\overline{\varepsilon }}_{1} } & {\dot{\overline{\varepsilon }}_{2} } & {\dot{\overline{\varepsilon }}_{3} } & {\dot{\overline{\varepsilon }}_{4} } & {\dot{\overline{\varepsilon }}_{5} } & {\dot{\overline{\varepsilon }}_{6} } \\ \end{array} } \right)^{{\text{T}}}$$, $${\overline{\mathbf{\varepsilon }}} = \left( {\begin{array}{*{20}c} {\overline{\varepsilon }_{1} } & {\overline{\varepsilon }_{2} } & {\overline{\varepsilon }_{3} } & {\overline{\varepsilon }_{4} } & {\overline{\varepsilon }_{5} } & {\overline{\varepsilon }_{6} } \\ \end{array} } \right)^{{\text{T}}}$$, $${{\varvec{\upupsilon}}} = \left( {\begin{array}{*{20}c} {\upsilon_{1} } & {\upsilon_{2} } & {\upsilon_{3} } & {\upsilon_{4} } & 0 & 0 \\ \end{array} } \right)^{{\text{T}}}$$. $a^{\prime}_{ij}$, $b^{\prime}_{ij}$ and $\upsilon_{i} (i = 1,2,3,4)$ are listed in the Online Appendix B.

When the vibrating system reaches the stable synchronous state, the small parameters $\varepsilon = 0$ and $\dot{\varepsilon } = 0$. Thus, the synchronous condition of four ERs can be expressed as Eq. (12).

12 $$ \begin{gathered} T_{e01} = f_{1} p\omega_{0} + m_{0} r^{2} \omega_{0} \kappa_{1} ,\left| {T_{e01} } \right| \le T_{eN1} \hfill \\ T_{e02} = f_{2} q\omega_{0} + m_{0} r^{2} \omega_{0} \kappa_{2} ,\left| {T_{e02} } \right| \le T_{eN2} \hfill \\ T_{e03} = f_{3} q\omega_{0} + m_{0} r^{2} \omega_{0} \kappa_{3} ,\left| {T_{e03} } \right| \le T_{eN3} \hfill \\ T_{e04} = f_{4} p\omega_{0} + m_{0} r^{2} \omega_{0} \kappa_{4} ,\left| {T_{e04} } \right| \le T_{eN4} \hfill \\ \end{gathered} $$

where $T_{eNi} (i = 1,2,3,4)$ are respectively the rated electromagnetic torques of four inductor motors. Because of the stable synchronous state, the result of ${{\varvec{\upupsilon}}} = 0$ can be obtained. Take ${{\varvec{\upupsilon}}} = 0$ into Eq. (12), and then Eq. (13) can be acquired.

13 $$ {\mathbf{A}}\user2{\dot{\overline{\varepsilon }}} = {\mathbf{B}}\overline{\user2{\varepsilon }} $$

As shown in Eq. (11), because the matrix ***A*** is a non-singular matrix and the determinant $\left| {\mathbf{A}} \right| \ne 0$, the matrix **A** is invertible. Then, Eq. (13) can be expressed as Eq. (14).

14 $$ \user2{\dot{\overline{\varepsilon }}} = {\mathbf{D}}\overline{\user2{\varepsilon }} $$

where ${\mathbf{D = A}}^{{ - {1}}} {\mathbf{B}}$. Because of $\left| {\lambda {\mathbf{I}} - {\mathbf{D}}} \right|{\mathbf{ = }}{0}$, the characteristic equation of the matrix can be represented as

15 $$ \lambda^{6} + d_{1} \lambda^{5} + d_{2} \lambda^{4} + d_{3} \lambda^{3} + d_{4} \lambda^{2} + d_{5} \lambda + d_{6} = 0 $$

where $d_{j} (j = 1,2,3,4,5,6)$ and $\lambda$ are respectively coefficients and characteristic values of the characteristic equation.

When the characteristic equation meets the condition of Hurwitz criterion, the synchronous state of the vibrating system is stable. Otherwise, it is unstable.

## Design of the controlling system

### Design of the electromechanical coupling system

**Figure 3:**
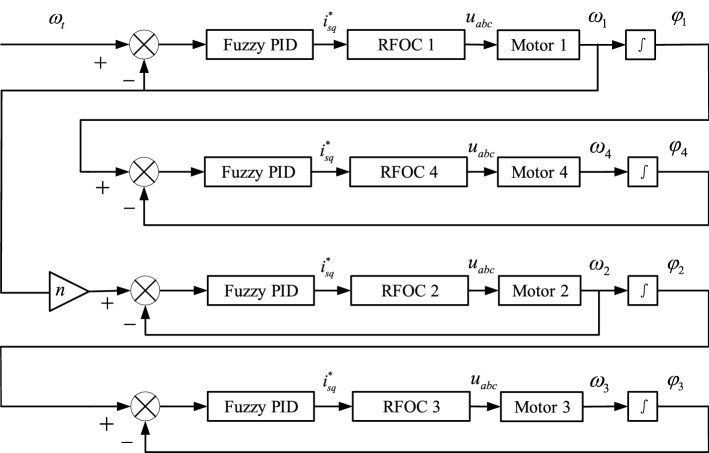
Framework diagram of the controlling system.

In this section, the controlling system of the vibrating system is shown as Fig. 3. The master–slave controlling strategy is introduced in the controlled structure and the fuzzy PID method is used in the vector controlling method of the inductor motors^26,27^. Motor 1 is considered as the master motor of the system. Motor 2 and 4 are both considered as the slave motors of motor 1. In the meanwhile, motor 3 is considered as the slave motor of motor 2.

To certify the feasibility of the controlling system, Fig. 3 should be illustrated. $\omega_{t}$ as a target speed is given firstly, and then through the fuzzy PID method, the speed of motor 1 $\omega_{1}$ can be obtained with RFOC 1. There are three functions of $\omega_{1}$. One is transferred to motor 1 as a feedback value. Another is given to motor 2 as an input value. The other is used to obtain $\varphi_{1}$ through the integral method. With the same frequency, the controlling system is traced through the phase, while it is traced through the speed with the fixed frequency ratio method. Thus, the speeds and phases of motor 2, 3 and 4 can be acquired.

### The stability analysis of the controlling system

Because there are two tracing situations in Fig. 3 which are respectively the speed tracing and phase tracing, the stability of the controlling system should be discussed separately. In the speed tracing situation, the speed of motor is set as the state variable, $\omega = \dot{\varphi }$. Taking $\omega = \dot{\varphi }$ into Eq. (4), thus Eq. (4) can be expressed as

16 $$ J_{i} \dot{\omega }_{i} + f\omega_{i} = K_{{T_{i} }} u_{i} + W_{i} (i = 1,2,3,4) $$

where $K_{Ti} = L_{mi} \phi_{rdi} /L_{ri} (i = 1,2,3,4)$, $u_{i}$ as the controlling variable represents $i_{qsi}^{ * }$, $i = 1,2,3,4$. $W_{i} = - T_{Li} (i = 1,2,3,4)$ represents the uncertain loads. $J_{i} (i = 1,2,3,4)$, respectively represents the rotational inertia of four motors. Combined the given target speed $\omega_{t}$ in Fig. 3 with the actual speed $\omega$, the speed error of the motor can be expressed as

17 $$ e = \omega_{t} - \omega $$

Then, the tracing error of the system can be represented as ${\boldsymbol{E}} = [e,\dot{e}]^{{\text{T}}}$. According to Eq. (16), the control law of the system can be designed as

18 $$ u = J/K_{T} [ - \hat{f}(x|\theta_{f} ) + \dot{\omega }_{t} + {\boldsymbol{K}}^{{\text{T}}} {\boldsymbol{E}} + (f\omega - W)/J] $$

where ${\boldsymbol{K}} = [k_{p} ,k_{i} ]^{{\text{T}}}$. The function $\hat{f}(x)$ can be expressed as $\hat{f}(x|\theta_{f} ) = \theta_{f}^{{\text{T}}} \xi (x)$ which is based on the weight coefficient $\theta_{f}$. Thus, the adaptive law of controlling system can be designed as Eq. (19).

19 $$ \dot{\theta }_{f} = - \gamma {\boldsymbol{E}}^{{\text{T}}} {\mathbf{P}}{\boldsymbol{b}}\xi ({\boldsymbol{x}}) $$

where **P** is a positive definite matrix. Considering $\Omega_{f}$ as a convex set to assure the optimal weight coefficient $\theta_{f}^{ * }$ belongs to it and the weight coefficient $\theta_{f}$ is bounded. And then, $\theta_{f}^{*}$ can be structured as

20 $$ \theta_{f}^{*} = \arg \mathop {\min }\limits_{{\theta_{f} \in \Omega_{f} }} [\sup |\hat{f}(x|\theta_{f} ) - f(x)] $$

Taking Eq. (18) into Eq. (16), the closed loop equation of the controlling system is designed as

21 $$ {\dot{\boldsymbol{E}}} = {{\varvec{\Lambda}}}{\boldsymbol{E}} + {\boldsymbol{b}}[\hat{f}(x|\theta_{f} ) - f(x)] $$

where $${\mathbf{b}} = \left( \begin{gathered} 0 \hfill \\ 1 \hfill \\ \end{gathered} \right)$$, $${{\varvec{\Lambda}}} = \left( {\begin{array}{*{20}c} 0 & 1 \\ { - k_{p} } & { - k_{i} } \\ \end{array} } \right)$$.

As illuminated in section "Design of the electromechanical coupling system", because the controlling system is a feedback system, the speed error and phase error should be considered. Thus, Eq. (21) can be converted to Eq. (22) which is the approximate error equation with Eqs. (18) and (19).

22 $$ \dot{\boldsymbol{E}} = {{\varvec{\Lambda}}}{\boldsymbol{E}} + {\boldsymbol{b}}[(\theta_{f} - \theta_{f}^{*} )^{{\text{T}}} \xi (x) + \Gamma ] $$

where $\Gamma = \hat{f}(x|\theta_{f}^{*} ) - f(x)$ is the minimum approximate error.

To acquire the minimum values of ***E*** and $\theta_{f} - \theta_{f}^{*}$, a Lyapunov function is constructed as Eq. (23).

23 $$ {\boldsymbol{V}} = {\boldsymbol{E}}^{\text{T}} {\mathbf{P}}{\boldsymbol{E}}/2 + (\theta_{f} - {\theta_{f}^{*}} )^{{\text{T}}} (\theta_{f} - {\theta_{f}^{*}} )/(2\zeta ) $$

where $\zeta$ is a positive real number. **Q** as a positive definite matrix is introduced to satisfy the stability of the Lyapunov equation.

24 $$ {{\varvec{\Lambda}}}^{{\text{T}}} {\mathbf{P}} + {\mathbf{PA}} = - {\mathbf{Q}} $$

Set $V_{1} = E^{{\text{T}}} {\mathbf{P}}E/2$, the derivative $\dot{V}_{{1}} = - E^{{\text{T}}} {\mathbf{Q}}E/2 + (\theta_{f} - \theta_{f}^{*} )^{{\text{T}}} E^{{\text{T}}} {\mathbf{P}}b\xi (x) + E^{{\text{T}}} {\mathbf{P}}b\Gamma$. Similarly, set ${\mathbf{V}}_{2} = (\theta_{f} - \theta_{f}^{*} )^{{\text{T}}} (\theta_{f} - \theta_{f}^{*} )/(2\zeta )$, and then the derivative ${\dot{\mathbf{V}}}_{2} = (\theta_{f} - \theta_{f}^{*} )^{{\text{T}}} \dot{\theta }_{f} /\zeta$. Based on the Lyapunov criterion, the derivative of the equation can be expressed as ${\dot{\mathbf{V}}} = {\dot{\mathbf{V}}}_{{1}} + {\dot{\mathbf{V}}}_{{2}}$, bring ${\mathbf{V}}_{1}$ and ${\mathbf{V}}_{2}$ into ${\mathbf{V}}$, it can be derived as $\dot{V} = - E^{{\text{T}}} {\mathbf{Q}}E/2 + E^{{\text{T}}} {\mathbf{P}}b\Gamma$. When the values of $\Gamma$ which can satisfy the condition ${\dot{\mathbf{V}}} \le 0$ exist, the system is stable.

With the same method above, the stability certification of speed error and phase error with the other motors can be obtained.

## Numerical and experimental results with discussions

In this section, some representative numerical simulation examples are given. The results show that demonstrate that the multifrequency self-synchronization can’t be realized. However, the multifrequency controlled synchronization can be realized through the fuzzy PID method. And then, the same results are given with the experiments. The parameters in the simulations and experiments are listed in Tables 2 and 3.

**Table 2:** The parameters in the vibration system.

Parameters	Values
*M*/kg	304
*J*_*p*_/(kg.m^2^)	44.5
*k*_*x*_/(N/m)	129,332
*k*_*y*_/(N/m)	105,334
*k*_*ψ*_/(Nm/rad)	30,715
*f*_*x*_ /(Ns/m)	615.5
*f*_*y*_/(Ns/m)	618
*f*_*ψ*_/(Nsm/rad)	180.2
*θ*_1_, *θ*_2_, *θ*_3_ *θ*_4_ /(°)	30, 150, 210, 330
*m*_0_/kg	6
*r*/m	0.05
*l*_1_, *l*_2_,* l*_3_,* l*_4_ /m	0.45, 0.45, 0.45, 0.45

**Table 3:** The parameters of four motors.

Parameters	Motor 1	Motor 2	Motor 3	Motor 4
*P*/kW	0.2	0.2	0.2	0.2
*n*_*p*_	3	3	3	3
*f*_0_/Hz	50	50	50	50
*U*/V	220	220	220	220
*n*/(r/min)	950	950	950	950
*R*_*s*_/Ω	40.5	40.5	40.5	40.5
*R*_*r*_*/*Ω	12	12	12	12
*L*_*s*_/H	1.21275	1.21275	1.2175	1.21275
*L*_*r*_/H	1.222	1.222	1.225	1.222
*L*_*m*_/H	1.116	1.116	1.116	1.116
$\lambda_{dr}^{*}$/Wb	0.98	0.98	0.98	0.98
*f*_1,2,3,4_/(Nms/rad)	0.005	0.005	0.005	0.005

### Results of numerical simulation

**Figure 4:**
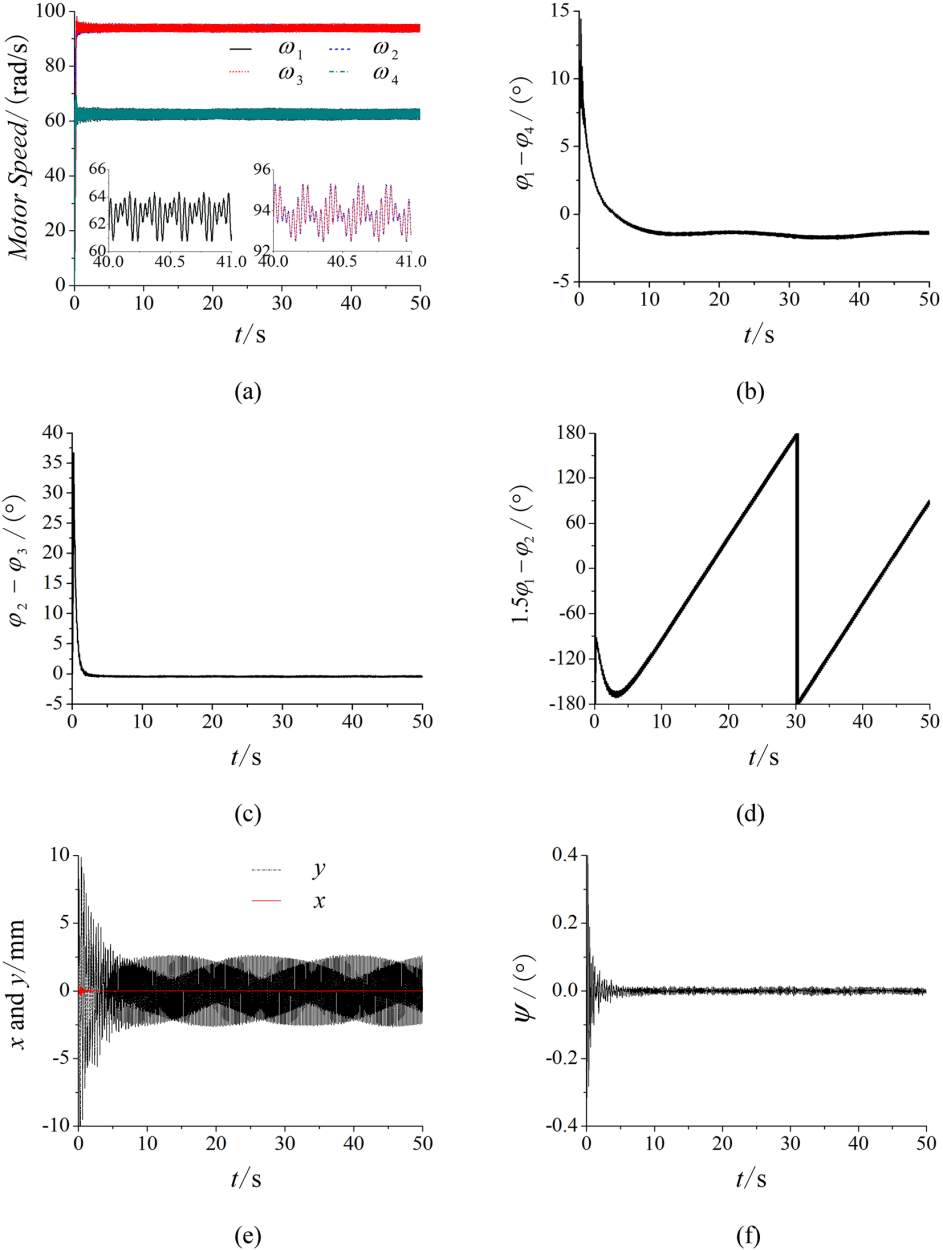
Multifrequency self-synchronization with four ERs, $\alpha_{0} { = }0$, *n* = 1.5. (**a**) Speed, (**b**) phase difference between motor 1 and 4, (**c**) phase differences between motor 2 and 3, (**d**) phase differences between motor 1 and 2, (**e**) response in the *x* and *y* direction, (**f**) response in the *ψ* direction.

Based on the model of Fig. 1, the frequency of motor 1 and 4 are set as 30 Hz and the frequency of motor 2 and 3 are both 45 Hz. The simulation results in Fig. 4a represent that the speeds of four motors reach the given speeds. From Fig. 4b, it represents that the phase between motor 1 and 4 approximately equals to zero. This result shows that two motors rotated in a opposite direction with the same frequency can reach the stable synchronization state. Similarly, the phase between motor 2 and 3 also trends to zero in Fig. 4c. With the non-linear theory, when the system can achieve $p\dot{\varphi }_{1} - q\dot{\varphi }_{2}$ = 0 and $p\varphi_{1} - q\varphi_{2}$ = constant, the multifrequency self-synchronization can be realized. However, the phase difference in Fig. 4d is a monotonic curve and $p\varphi_{1} - q\varphi_{2} \ne$ constant. This result represents that the multifrequency self-synchronization of the vibration system can’t be realized. Figure 4e,f are respectively the responses in three directions. The results in Fig. 4 are consistent with the dynamical model.

**Figure 5:**
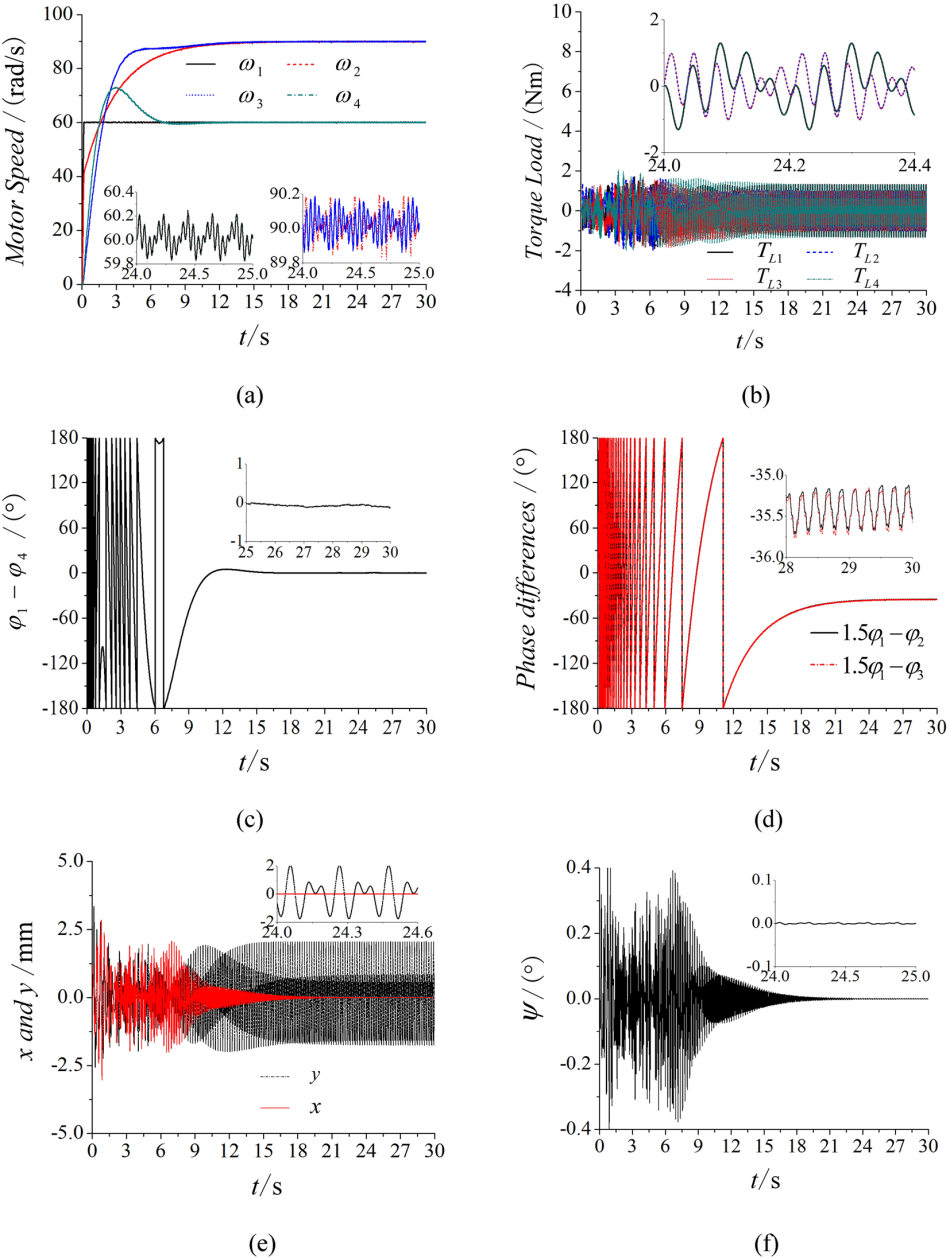
Controlled synchronization with four ERs, $\alpha_{0} { = }0$, *n* = 1.5, $\eta = 50\%$. (**a**) Speeds, (**b**) load torques, (**c**) phase difference between motor 1 and 4, (**d**) phase differences between motor 1 with motor 2 and 3, (**e**) responses in the *x* and y direction, (**f**) response in the *ψ* direction.

**Figure 6:**
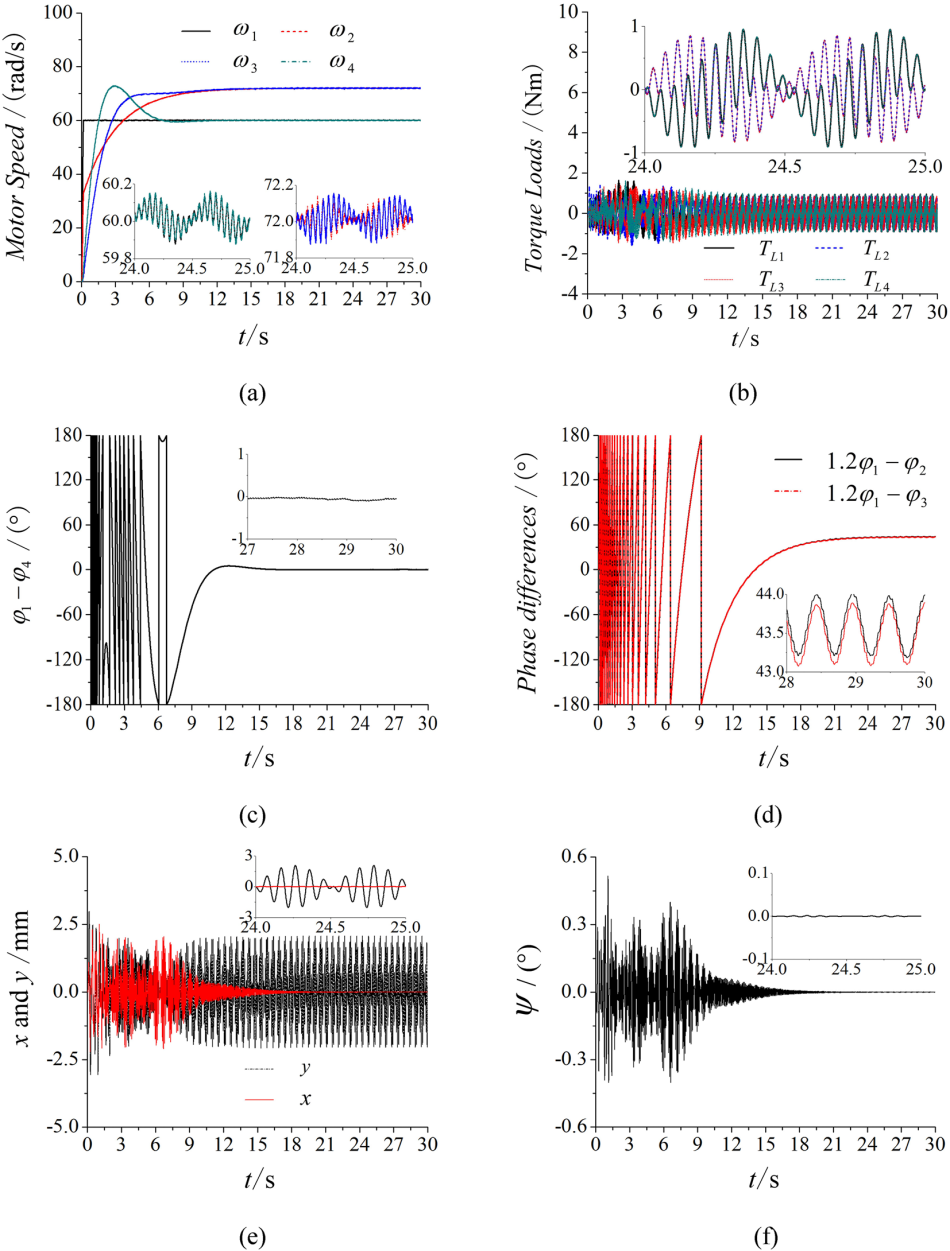
Controlled synchronization with four ERs, $\alpha_{0} { = }0$, *n* = 1.2, $\eta = 50\%$. (**a**) Speeds, (**b**) load torques, (**c**) phase difference between motor 1 and 4, (**d**) phase differences between motor 1 with motor 2 and 3, (**e**) responses in the *x* and y direction, (**f**) response in the *ψ* direction.

With the results above, the multifrequency self-synchronization can’t be realized. Thus, the fixed frequency ratio method is introduced in the vibrating system. As shown in Fig. 5a is the speeds of four motors. The given speed of motor 1 is 60 rad/s, the speeds of motor 2, 3 and 4 respectively attain 90 rad/s, 90 rad/s and 60 rad/s with the controlling method. Figure 5b is the torque load of four inductor motors. The values of torque loads are between − 2 and 2, so the phenomenon of locked-rotor and overload with motors can’t appear. All the inductor motors can operate smoothly. In Fig. 5c, the phase between motor 1 and 4 nearly equals zero, which illustrates motor 1 and 4 reach the stable synchronous state. Figure 5d is phase differences between motor 1 with motor 2 and 3. The phase differences both equal to constants. This result shows that the controlled synchronization is realized with the fixed frequency ratio method. Figure 5e,f are responses in the three directions. In Fig. 5e, two groups of motors both rotate in the opposite direction. Thus, the forces in the *y* direction counteract with each other and the amplitude in the *y* direction is nearly to zero, while the amplitudes in the *x* direction are superimposed. This result accords with the proposed dynamical theory. In Fig. 5f, the response in the *ψ* direction tends to zero. This phenomenon shows that there is no swing in the vibrating system. Through the Fig. 5, it can be known that the vibrating system reaches the stable synchronous state and the controlled synchronization is realized with the fixed frequency ratio method. To guarantee the arbitrariness of the parameter *n*, another simulation of which the parameter is altered from 1.5 to 1.2 is presented in Fig. 6. In Fig. 6, the torque loads of four motors still satisfy the operation requirements based on the values between − 1 and 1. Through the speeds and phase differences in Fig. 6a,c,d, the results represent that the vibrating system can realize the controlled synchronization with the parameter *n* = 1,2. Although the response of the *y* direction in Fig. 6e is different from the response in Fig. 5 because of the different parameter *n*, the responses of three directions are still consistent with the dynamical model. So, the vibrating system can realize the stable controlled synchronization. This result demonstrates the arbitrariness of the parameter only if conditions of torque loads are satisfied.

### Results of experimental verification

**Figure 7:**
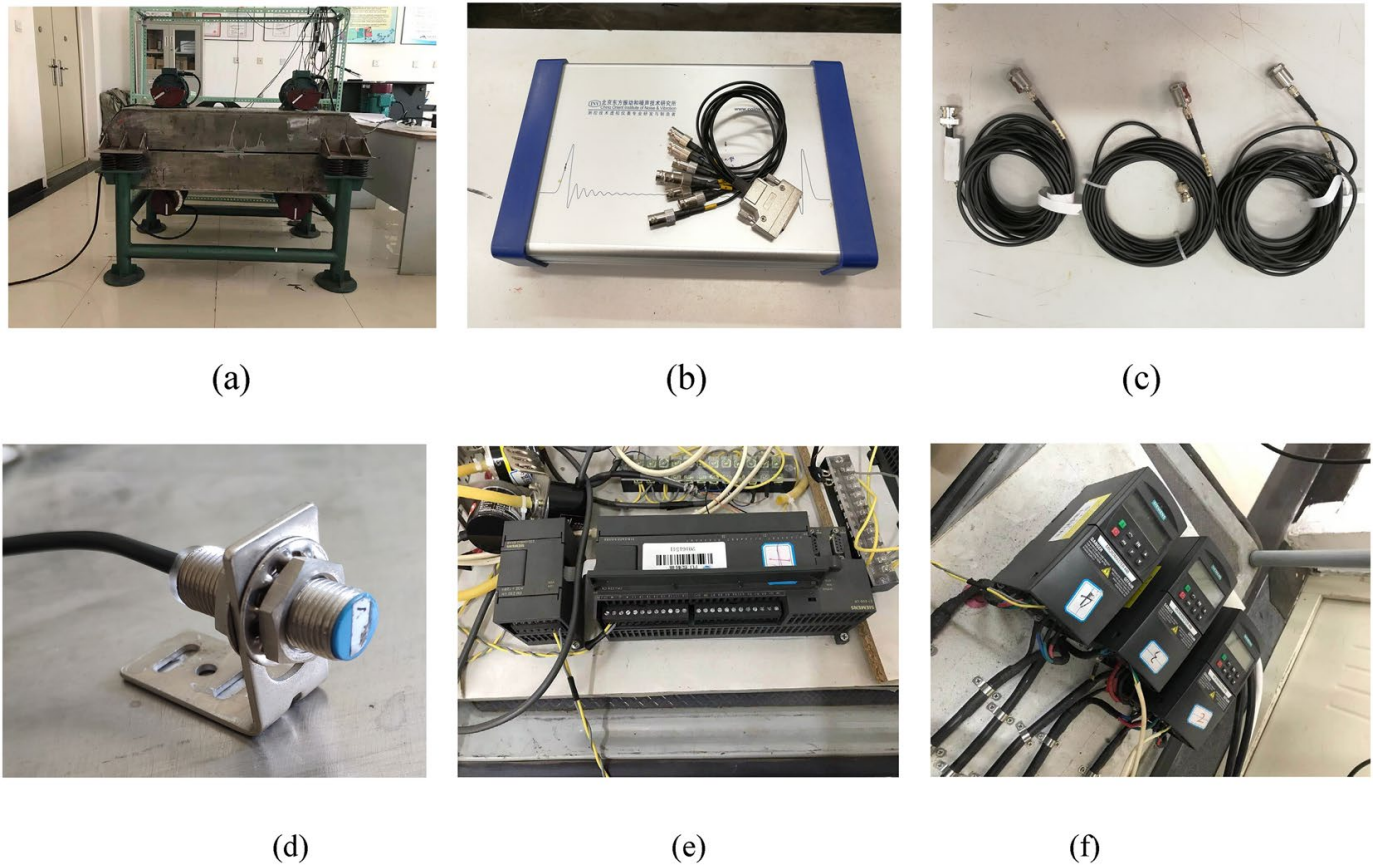
Experiment equipment of the vibrating system. (**a**) The vibrating testing bench, (**b**) the data acquisition and signal processing, (**c**) the acceleration sensors, (**d**) the Hall magnetic switch, (**e**) the Programmable Logic controller, (**f**) the convertors.

**Figure 8:**
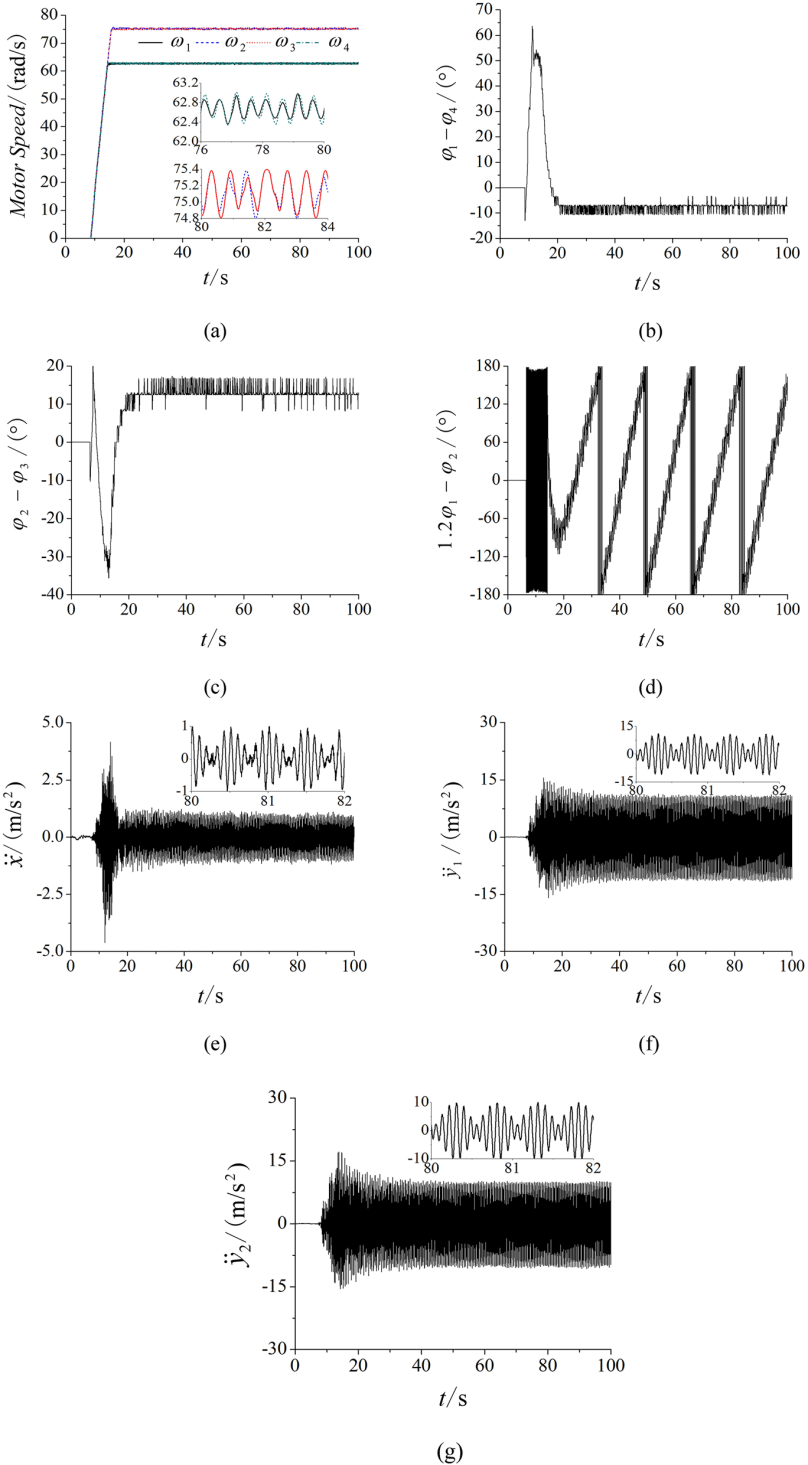
Experiment of multifrequency self-synchronization with four ERs, $\alpha_{0} { = }0$, *n* = 1.2, $\eta = 50\%$. (**a**) Speeds, (**b**) phase difference between motor 1 and 4, (**c**) phase differences between motor 2 and 3, (**d**) phase differences between motor 1 and 2, (**e**) response in the *x* direction, (**f**) response in the *y*_1_ direction, (**g**) response in the *y*_2_ direction.

To validate the accuracy of the theory proposed, the main experiment equipment are listed in Fig. 7 firstly. The frequencies of four inductor motors are set by four convertors of which type are Siemens MM440. The PLC (Programmable Logic controller) are connected with the convertors. The three acceleration sensors are sticked on the vibrating testing bench and connected with the DASP (Data acquisition & signal processing) of which another port is connected with a computer. And then, the experiment of multifrequency self-synchronization is given based on *n* = 1.2 to comparison with the simulation in Fig. 4. As shown in Fig. 8, the frequencies of four motors are respectively set as 30 Hz, 36 Hz, 36 Hz and 30 Hz. From (a), the speeds are stable and reach preset value. Although the difference in (b) is between − 5 and − 12, motor 1 and 4 can be recognized to realize the self-synchronization motion due to the experiment error. Similar result is in figure (c). Compared with the result in (d) of Fig. 4, (d) in Fig. 8 shows the same result that the curve of the phase difference between motor 1 and 2 is also monotonous. So, the multifrequency self-synchronization can’t be realized. The responses of three directions in (e) and (f) are in accordance with the simulation and theory result. This experiment indicates that the multifrequency self-synchronization can’t be realized whatever *n* equals to.

**Figure 9:**
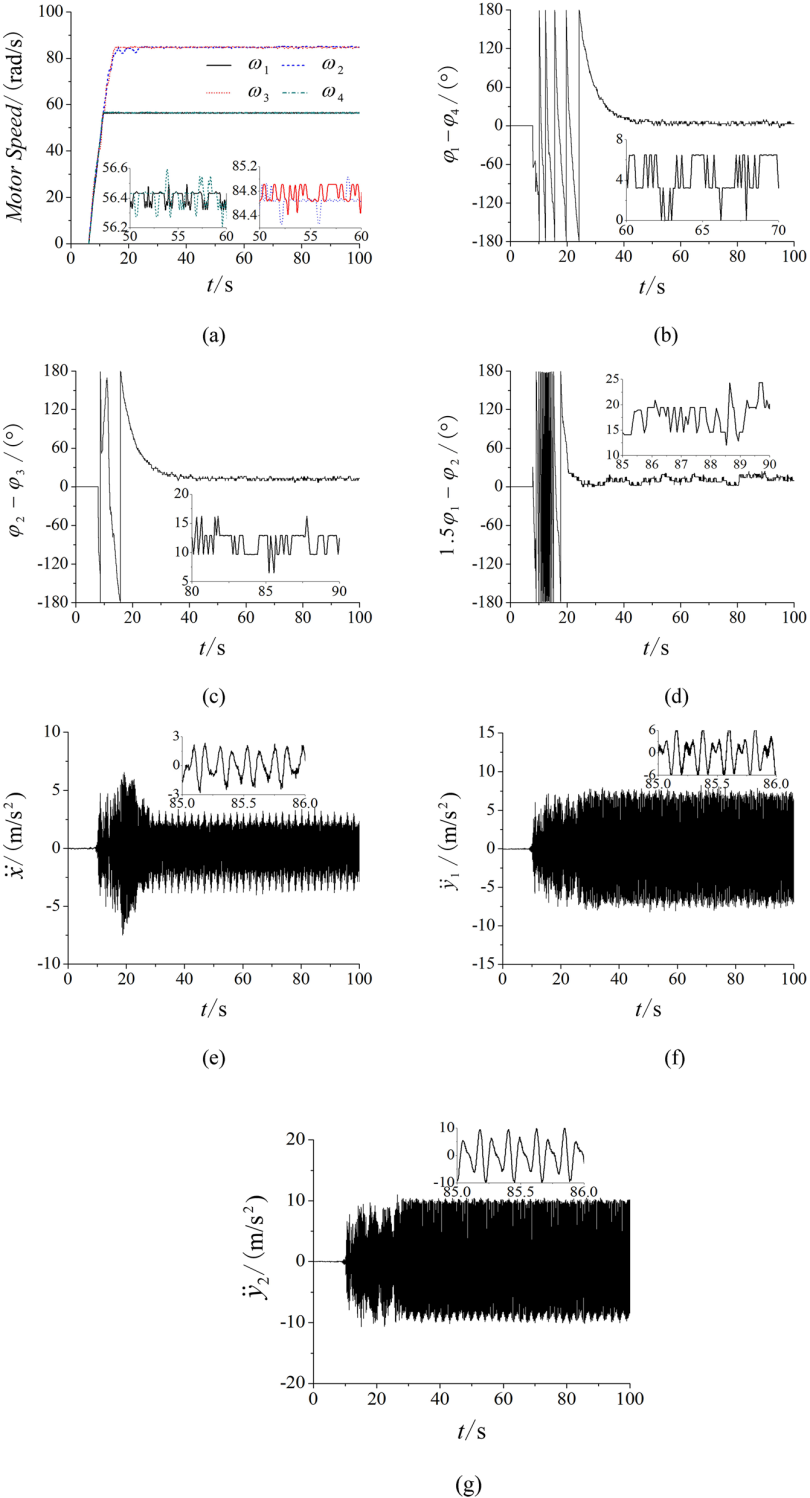
Experiment of controlled synchronization with four ERs, $\alpha_{0} { = }0$, *n* = 1.5, $\eta = 50\%$. (**a**) speeds, (**b**) phase difference between motor 1 and 4, (**c**) phase differences between motor 2 and 3, (**d**) phase differences between motor 1 and 2, (**e**) response in the *x* direction, (**f**) response in the *y*_1_ direction, (**g**) response in the *y*_2_ direction.

In Fig. 9, the experiment of controlled synchronization is illustrated based on the parameter *n* = 1.5. From Fig. 9a–c, motor 1 with 4 and motor 2 with 3 both realize the controlled synchronization. In Fig. 9d, the phase difference between motor 1 and 2 trends to a constant, which confirms that the controlled synchronization is realized. In Fig. 9e, the response is obviously smaller than the response in Fig. 9g, so this result indicates that the vibrating system realize the stable synchronous state. The response curve of (e) in Fig. 9 is similar with the curve of (e) in Fig. 5 and this experimental result is correspond with the simulation.

## Conclusions

The article investigates the multifrequency controlled synchronization of four inductor motors with the fixed frequency ratio method based on a mass-spring rigid body. Through the dynamical model is derived, the stability and synchronization conditions of the vibrating system are both obtained. This result indicates that although the self-synchronization with the same frequency can be realized, the multifrequency self-synchronization of vibrating system can’t be realized in the dynamical model of Fig. 1. Some numerical simulations and experiments are given to certify the consistency of the result. Via introducing the proposed the fixed frequency ratio method in the controlling system, the multifrequency controlled synchronization is realized. Through the robustness analysis, the stability of the controlling system is certified to illuminate the feasibility of the controlling method. The conformity of the theory by the simulations and experiments is illustrated. The result indicates that only if the conditions of torque loads can be satisfied, the arbitrariness of the fixed frequency parameter can be realized with the proposed method. Additionally, the multifrequency controlled synchronization method provides a novel way to address the problem of multifrequency vibrating screen in the industry.

## Supplementary Information

Supplementary Information.

## Data Availability

The datasets generated during the current study are not publicly available until the project funding in this article is finished, but are available from the corresponding author on reasonable request.
