# Supplementary material for: Multifrequency controlled synchronization of four inductor motors by the fixed frequency ratio method in a vibration system
Source: Sci Rep. 2023 Feb 11;13:2467. doi: 10.1038/s41598-023-29603-y (PMC9922278; doi:10.1038/s41598-023-29603-y)
Supplement: Supplementary file 1 — Supplementary Information. [file 41598_2023_29603_MOESM1_ESM.pdf]

Multifrequency controlled synchronization of four inductor motors by the fixed frequency ratio method in a vibration system

Lei Jia*[1,2], Chun Wang[1], Ziliang Liu[1]

[1]School of Mechanical Engineering, Shenyang Ligong University, Shenyang 110159, China.

[2]Shenyang Ligong University, Science and Technology Development Corporation, Shenyang 110159, China.

*Corresponding author. Email: jialeizsq@126.com

Appendix A

The coefficient and constant items in equation (10).

$$a_{11} = -\eta_1^2[r_m \cos\gamma_{x1} / \mu_{x1} + r_m \cos\gamma_{y1} / \mu_{y1} + r_m r_{l1}^2 \cos\gamma_{\psi1} / \mu_{\psi1}] / 2 \quad \text{(A.1)}$$

$$\begin{aligned} a_{14} = \eta_1\eta_4[&r_m \cos(2\alpha_1 - \gamma_{x4}) / \mu_{x4} - r_m \cos(2\alpha_1 - \gamma_{y4}) / \mu_{y4} \\ &- r_m r_{l1} r_{l4} \cos(2\alpha_1 - \theta_4 - \theta_1 - \gamma_{\psi4}) / \mu_{\psi4}] / 2 \end{aligned} \quad \text{(A.2)}$$

$$b_{11} = \eta_1^2\omega_0 p[r_m \sin\gamma_{x1} / \mu_{x1} + r_m \sin\gamma_{y1} / \mu_{y1} + r_m r_{l1}^2 \sin\gamma_{\psi1} / \mu_{\psi1}] \quad \text{(A.3)}$$

$$\begin{aligned} b_{14} = \eta_1\eta_4\omega_0 p[&r_m \sin(2\alpha_1 - \gamma_{x4}) / \mu_{x4} - r_m \sin(2\alpha_1 - \gamma_{y4}) / \mu_{y4} \\ &- r_m r_{l1} r_{l4} \sin(2\alpha_1 - \theta_4 - \theta_1 - \gamma_{\psi4}) / \mu_{\psi4}] \end{aligned} \quad \text{(A.4)}$$

$$a_{22} = -\eta_2^2[r_m \cos\gamma_{x2} / \mu_{x2} + r_m \cos\gamma_{y2} / \mu_{y2} + r_m r_{l2}^2 \cos\gamma_{\psi2} / \mu_{\psi2}] / 2 \quad \text{(A.5)}$$

$$\begin{aligned} a_{23} = \eta_2\eta_3[&r_m \cos(2\alpha_2 - \gamma_{x3}) / \mu_{x3} - r_m \cos(2\alpha_2 + \gamma_{y3}) / \mu_{y3} \\ &- r_m r_{l2} r_{l3} \cos(2\alpha_2 - \theta_2 - \theta_3 - \gamma_{\psi3}) / \mu_{\psi3}] / 2 \end{aligned} \quad \text{(A.6)}$$

$$b_{22} = \eta_2^2\omega_0 q[r_m \sin\gamma_{x2} / \mu_{x2} + r_m \sin\gamma_{y2} / \mu_{y2} + r_m r_{l2}^2 \sin\gamma_{\psi2} / \mu_{\psi2}] \quad \text{(A.7)}$$

$$\begin{aligned} b_{23} = \eta_2\eta_3\omega_0 q[&r_m \sin(2\alpha_2 - \gamma_{x3}) / \mu_{x3} - r_m \sin(2\alpha_2 - \gamma_{y3}) / \mu_{y3} \\ &- r_m r_{l2} r_{l3} \sin(2\alpha_2 - \theta_2 - \theta_3 - \gamma_{\psi3}) / \mu_{\psi3}] \end{aligned} \quad \text{(A.8)}$$

$$\begin{aligned} a_{32} = \eta_2\eta_3[&r_m \cos(2\alpha_2 + \gamma_{x2}) / \mu_{x2} - r_m \cos(2\alpha_2 + \gamma_{y2}) / \mu_{y2} \\ &- r_m r_{l2} r_{l3} \cos(2\alpha_2 - \theta_2 - \theta_3 + \gamma_{\psi2}) / \mu_{\psi2}] / 2 \end{aligned} \quad \text{(A.9)}$$

$$a_{33} = -\eta_3^2[r_m \cos\gamma_{x3} / \mu_{x3} + r_m \cos\gamma_{y3} / \mu_{y3} + r_m r_{l3}^2 \cos\gamma_{\psi3} / \mu_{\psi3}] / 2 \quad \text{(A.10)}$$

$$\begin{aligned} b_{32} = -\eta_2\eta_3\omega_0 q[&r_m \sin(2\alpha_2 + \gamma_{x2}) / \mu_{x2} - r_m \sin(2\alpha_2 + \gamma_{y2}) / \mu_{y2} \\ &- r_m r_{l2} r_{l3} \sin(2\alpha_2 - \theta_2 - \theta_3 + \gamma_{\psi2}) / \mu_{\psi2}] \end{aligned} \quad \text{(A.11)}$$

$$b_{33} = \eta_3^2\omega_0 q[r_m \sin\gamma_{x3} / \mu_{x3} + r_m \sin\gamma_{y3} / \mu_{y3} + r_m r_{l3}^2 \sin\gamma_{\psi3} / \mu_{\psi3}] \quad \text{(A.12)}$$

$$\begin{aligned} a_{41} = \eta_1\eta_4[&r_m \cos(2\alpha_1 + \gamma_{x1}) / \mu_{x1} - r_m \cos(2\alpha_1 + \gamma_{y1}) / \mu_{y1} \\ &- r_m r_{l1} r_{l4} \cos(2\alpha_1 - \theta_4 - \theta_1 + \gamma_{\psi1}) / \mu_{\psi1}] / 2 \end{aligned} \quad \text{(A.13)}$$

$$a_{44} = \eta_4^2[r_m \cos\gamma_{x4} / \mu_{x4} + r_m \cos\gamma_{y4} / \mu_{y4} + r_m r_{l4}^2 \cos\gamma_{\psi4} / \mu_{\psi4}] / 2 \quad \text{(A.14)}$$

$$\begin{aligned} b_{41} = -\eta_1\eta_4\omega_0 p[&r_m \sin(2\alpha_1 + \gamma_{x1}) / \mu_{x1} - r_m \sin(2\alpha_1 + \gamma_{y1}) / \mu_{y1} \\ &- r_m r_{l1} r_{l4} \sin(2\alpha_1 - \theta_4 - \theta_1 + \gamma_{\psi1}) / \mu_{\psi1}] \end{aligned} \quad \text{(A.15)}$$

$$b_{44} = \eta_4^2\omega_0 p[r_m \sin\gamma_{x4} / \mu_{x4} + r_m \sin\gamma_{y4} / \mu_{y4} + r_m r_{l4}^2 \sin\gamma_{\psi4} / \mu_{\psi4}] \quad \text{(A.16)}$$

$$\kappa_1 = \frac{1}{2} p^2 \omega_0 [\eta_1^2 (r_m \sin\gamma_{x1} / \mu_{x1} + r_m \sin\gamma_{y1} / \mu_{y1} + r_m r_{l1}^2 \sin\gamma_{\psi 1} / \mu_{\psi 1}) + \eta_1 \eta_4 (r_m \sin(2\alpha_1 - \gamma_{x4}) / \mu_{x4} - r_m \sin(2\alpha_1 - \gamma_{y4}) / \mu_{y4} - r_m r_{l1} r_{l4} \sin(2\alpha_1 - \theta_4 - \theta_1 - \gamma_{\psi 4}) / \mu_{\psi 4})] \tag{A.17}$$

$$\kappa_2 = \frac{1}{2} q^2 \omega_0 [\eta_2^2 (r_m \sin\gamma_{x2} / \mu_{x2} + r_m \sin\gamma_{y2} / \mu_{y2} + r_m r_{l2}^2 \sin\gamma_{\psi 2} / \mu_{\psi 2}) + \eta_2 \eta_3 (r_m \sin(2\alpha_2 - \gamma_{x3}) / \mu_{x3} - r_m \sin(2\alpha_2 - \gamma_{y3}) / \mu_{y3} - r_m r_{l2} r_{l3} \sin(2\alpha_2 - \theta_2 - \theta_3 - \gamma_{\psi 3}) / \mu_{\psi 3})] \tag{A.18}$$

$$\kappa_3 = \frac{1}{2} q^2 \omega_0 [-\eta_2 \eta_3 (r_m \sin(2\alpha_2 + \gamma_{x2}) / \mu_{x2} - r_m \sin(2\alpha_2 + \gamma_{y2}) / \mu_{y2} - r_m r_{l2} r_{l3} \sin(2\alpha_2 - \theta_2 - \theta_3 + \gamma_{\psi 2}) / \mu_{\psi 2}) + \eta_3^2 (r_m \sin\gamma_{x3} / \mu_{x3} + r_m \sin\gamma_{y3} / \mu_{y3} + r_m r_{l3}^2 \sin\gamma_{\psi 3} / \mu_{\psi 3})] \tag{A.19}$$

$$\kappa_4 = \frac{1}{2} p^2 \omega_0 [-\eta_1 \eta_4 (r_m \sin(2\alpha_1 + \gamma_{x1}) / \mu_{x1} - r_m \sin(2\alpha_1 + \gamma_{y1}) / \mu_{y1} - r_m r_{l1} r_{l4} \sin(2\alpha_1 - \theta_4 - \theta_1 + \gamma_{\psi 1}) / \mu_{\psi 1}) + \eta_4^2 (r_m \sin\gamma_{x4} / \mu_{x4} + r_m \sin\gamma_{y4} / \mu_{y4} + r_m r_{l4}^2 \sin\gamma_{\psi 4} / \mu_{\psi 4})] \tag{A.20}$$

Appendix B

$$a'_{11}=\eta_1+a_{11},\ \ a'_{14}=a_{14},\ \ b'_{11}=-(f_1/m_0r^2+k_{e01}/m_0r^2\omega_0+b_{11}),\ \ b'_{14}=-b_{14} \quad \text{(B.1-B.4)}$$

$$\begin{aligned}b'_{15}=\eta_1\eta_4\omega_0 p[&r_m\cos(2\alpha_1-\gamma_{x4})/\mu_{x4}-r_m\cos(2\alpha_1-\gamma_{y4})/\mu_{y4}\\&-r_mr_{l1}r_{l4}\cos(2\alpha_1-\theta_4-\theta_1-\gamma_{\psi 4})/\mu_{\psi 4}]\end{aligned} \quad \text{(B.5)}$$

$$a'_{22}=\eta_2+a_{22},\ \ a'_{23}=a_{23},\ \ b'_{22}=-(f_2/m_0r^2+k_{e02}/m_0r^2\omega_0+b_{22}),\ \ b'_{23}=-b_{23} \quad \text{(B.6-B.9)}$$

$$\begin{aligned}b'_{26}=\eta_2\eta_3\omega_0 q[&r_m\sin(2\alpha_2-\gamma_{x3})/\mu_{x3}-r_m\sin(2\alpha_2-\gamma_{y3})/\mu_{y3}\\&-r_mr_{l2}r_{l3}\sin(2\alpha_2-\theta_2-\theta_3-\gamma_{\psi 3})/\mu_{\psi 3}]\end{aligned} \quad \text{(B.10)}$$

$$a'_{32}=a_{32},\ \ a'_{33}=\eta_3+a_{33},\ \ b'_{32}=-b_{32},\ \ b'_{33}=-(f_3/m_0r^2+k_{e03}/m_0r^2\omega_0+b_{33}) \quad \text{(B.11-B.14)}$$

$$\begin{aligned}b'_{36}=-\eta_2\eta_3\omega_0 q[&r_m\cos(2\alpha_2+\gamma_{x2})/\mu_{x2}-r_m\cos(2\alpha_2+\gamma_{y2})/\mu_{y2}\\&-r_mr_{l2}r_{l3}\cos(2\alpha_2-\theta_3-\theta_2+\gamma_{\psi 2})/\mu_{\psi 2}]\end{aligned} \quad \text{(B.15)}$$

$$a'_{41}=a_{41},\ \ a'_{44}=\eta_4+a_{44},\ \ b'_{41}=-b_{41},\ \ b'_{44}=-(f_4/m_0r^2+k_{e04}/m_0r^2\omega_0+b_{44}) \quad \text{(B.16-B.19)}$$

$$\begin{aligned}b'_{45}=-\eta_1\eta_4\omega_0 p[&r_m\cos(2\alpha_1+\gamma_{x1})/\mu_{x1}-r_m\cos(2\alpha_1+\gamma_{y1})/\mu_{y1}\\&-r_mr_{l1}r_{l4}\cos(2\alpha_1-\theta_4-\theta_1+\gamma_{\psi 4})/\mu_{\psi 1}]\end{aligned} \quad \text{(B.20)}$$

$$\upsilon_1=T_{e01}/m_0r^2\omega_0-f_1p/m_0r^2-\kappa_1,\ \ \upsilon_2=T_{e02}/m_0r^2\omega_0-f_2p/m_0r^2-\kappa_2 \quad \text{(B.21-B.22)}$$

$$\upsilon_3=T_{e03}/m_0r^2\omega_0-f_3q/m_0r^2-\kappa_3,\ \ \upsilon_4=T_{e04}/m_0r^2\omega_0-f_4p/m_0r^2-\kappa_4 \quad \text{(B.23-B.24)}$$
